# Synthetic strategy towards novel composite based on substituted pyrido[2,1-*b*][1,3,4]oxadiazine-dialdehyde chitosan conjugate with antimicrobial and anticancer activities

**DOI:** 10.1186/s13065-023-01005-1

**Published:** 2023-07-26

**Authors:** Reham A. Mohamed-Ezzat, Amr H. Hashem, Sawsan Dacrory

**Affiliations:** 1 Chemistry of Natural and Microbial Products Department, Pharmaceutical and Drug Industries Research Institute, National Research Centre, Cairo, Egypt; 2 Botany and Microbiology Department, Faculty of Science, Al-Azhar University, Cairo, 11884 Egypt; 3 Cellulose and Paper Department, National Research Centre, 33 El Bohouth St, Giza, 12622 Egypt

**Keywords:** Synthesis, Pyrido[2,1-*b*][1,3,4]oxadiazine, Dialdehyde chitosan, Breast cancer, Anti-microbial activity

## Abstract

Synthesis of new compounds that have biological activity is an indispensible issue in order to deal with the drug resistant bacteria. This wok reports preparation of a novel composite based on substituted pyrido[2,1-*b*][1,3,4] oxadiazine-dialdehyde chitosan (PODACs) conjugate. Firstly, a novel approach of synthesizing of a new substituted pyrido[2,1-*b*][1,3,4]oxadiazine-7-carboxylic acid (PO) is reported through reacting(Z)-*N*’-(1-(3-aminophenyl)ethylidene)-2-cyanoacetohydrazide with (Z)-ethyl 2-cyano-3-(pyridin-3-yl)acrylate. Then Dialdehyde chitosan (DACs) has prepared via periodat oxidation of chitosan (Cs). The synthesized compounds have studied via various spectroscopic instruments to validate their chemical structure such as nuclear magnetic resonance 1 H NMR, ^13^ C NMR, fourier transform infrared spectroscopy (FTIR), X-ray powder diffraction (XRD), and scanning electron microscopy (SEM). The substituted pyrido [2,1-*b*][1,3,4]oxadiazine and the composite were evaluated for antimicrobial activity against pathogenic bacteria and unicellular fungi. The results revealed that, the composite exhibited promising antimicrobial activity against *E. coli*, *S. aureus, B. subtilis* and *C. albicans* where inhibition zones were 19, 18, 36 and 20 mm respectively. Furthermore, the substituted pyrido [2,1-*b*][1,3,4]oxadiazine and the composite were evaluated for cytotoxic activity against MCF-7 human breast cancer cell line as well as vero normal cell line. Results illustrated the prepared composite has anticancer activity against MCF7 where IC_50_ was 238 µg/ml.

**Supplementary Information:**

The online version contains supplementary material available at 10.1186/s13065-023-01005-1.

## Introduction

**Fig. 1:**
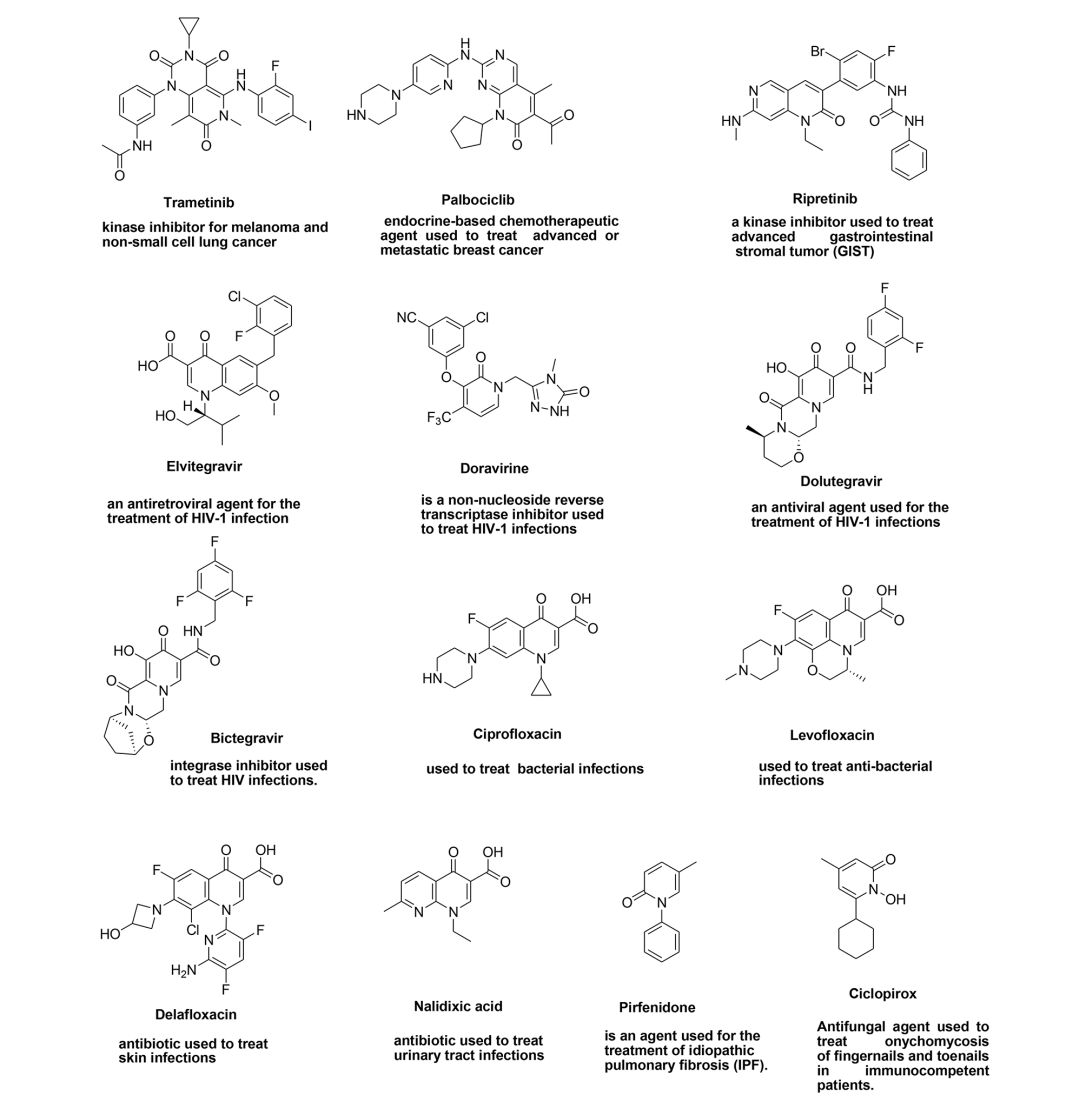
Pyridine containing drugs

Multi-drug resistant bacteria emerged due to the overuse or misuse of antibiotics; additionally, the modern treatment of antibiotics via clinically prescribed dosages is not able to manage these pathogens, and therefore preventive strategies are necessary [1]. Mortality of fungal pathogens becomes equal to drug-resistant Mycobacterium tuberculosis and exceeds that of malaria [2]. Drug-resistant microbes have been widely spread and have acquired new mechanisms to resist antibacterial and antifungal drugs. Additionally, Cancer is considered the second largest cause of death worldwide and has historically been one of the most common diseases with the highest mortality rates [3]. Previously, chemotherapy was used to treat cancer without a clear understanding of the target, protein, or enzyme responsible, leading to the inhibition of the entire family of enzymes or receptors and causing high levels of toxicity and side effects. Therefore, it is necessary to design and develop new compounds that overcome these limitations. Various pharmaceutical agents are considered as potent chemotherapeutic agents which have great potential impact on medical research were developed [4]. As part of our current researches in synthesizing various antimetabolites [5, 6], the substituted pyridone as a deaza pyrimidine analog has been designed and synthesized. Recently, 2-pyridones have integrated considerable significance as these compounds display various biological potencies (Fig. 1) such as antitumoral [7], antimalarial [8], analgesic [9] and anti-HIV [10] properties. Furthermore, 2-pyridones are a category of newly discovered active antibacterial agents that are particular interest due to their in vitro and in vivo antibacterial potencies against the bacterial type II DNA topoisomerases, which comprise topoisomerase IV and two highly homologous enzymes-DNA gyrase [11]. It is worthy to note that there is an increasing interest in techniques that allow conjugation of synthesized heterocylic compounds with biopolymers such as cellulose, chitosan, and sodium alginate. Due to the unique properties of these biopolymers such as biocompatibility, biodegradability, cost-effective and non-toxic, it opens new avenue in drug preparation approaches. Among these biopolymers, Chitosan(Cs) has interesting properties due to the existence of primary amines along its backbone chain which makes this polysaccharide an important candidate in the field of biomaterials [12]. In spite of its unique properties, some of its properties are not suitable for biomedical applications e.g. its solubility in water only at low pH. Thus modification of chitosan became essential process to enhance its properties. Functionalization of Cs with dialdehyde groups leads to ring opening formation via selective oxidation of C2-C3 by periodat oxidation [13]. This work elucidates the formation of new heterocyclic compound based on DACs as a biopolymer and the novel substituted PO. The prepared compounds are investigated via FTIR spectra, XRD, and SEM. Additionally, antimicrobial activity against pathogenic bacteria and unicellular fungi has studied. Cytotoxic activity against MCF-7 human breast cancer cell line and toward Vero normal cell line has evaluated.

## Experimental

### Materials

#### Chemical methods

Chitosan was purchased from sigma Aldrich, sodium periodate was purchased from Analytical Rasayan and starting materials were purchased from Aldrich, Acros, or Fluorochem and were utilized without purification. Reaction progress was monitored by TLC on pre-coated silica gel 60 F_245_aluminium plates with visualization under UV light. Determination of the melting point was carried out using open capillary tubes on a Stuart SMP30 melting point apparatus and was uncorrected. Spectral data of the compounds were carried out in the Micro-analytical labs in the National Research Centre Cairo, Egypt. The NMR spectra was measured on a Bruker Fourier 500 (at 500 MHz & 75 MHz, respectively) at 300 K. FT-IR spectra were recorded in the range of 400–4000 cm − 1on (Shimadzu 8400 S) FT-IR Spectrophotometer. The surface morphology was analyzed using (SEM) electron microscope FEI IN SPECTS Company, Philips, Poland, environmental scanning without coating with a JEOL JEM-2100 electron microscope at 100k x magnification and an acceleration voltage of 120 kV. Diano X-ray diffractometer was used for investigating XRD patterns using CuKα radiation source energized at 45 kV and a Philips X-ray diffractometer (PW 1930 generator, PW 1820 goniometer) with CuK radiation source (λ = 0.15418 nm), at a diffraction angle range of 2θ from 5 to 70° in reflection mode.

#### Synthesis of 6-amino-3-(3-aminophenyl)-9-cyano-2,9a-dihydro-8-(pyridin-3-yl)pyrido[2,1-***b***][1,3,4]oxadiazine-7-carboxylic acid (5)(PO)

A mixture of (Z)-N’-(1-(3-aminophenyl)ethylidene)-2-cyanoacetohydrazide (0.01 mol) and (Z)-ethyl 2-cyano-3-(pyridin-3-yl)acrylate (0.01 mol), was refluxed for 3 h in methanol / acetic acid (1:2). The precipitated solid product was filtered off and recrystallized from methanol. Compound **5** was afforded as a yellow solid (81%), m.p. 300–303 °C; ^1^ H NMR (500 MHz, DMSO-d_6_): 4.47 (s, 2 H, CH_2_), 4.95 (s, 1 H, CH), 7.35–7.42 (d, 4 H, CH), 7.64 (s, 1 H, CH), 8.37–8.46 (d, 3 H, CH), 11.00 (s, 5 H, OH, 2NH_2_). C ^13^ NMR (500 MHz, DMSO-*d*_6_): 39.64, 39.80, 40.13, 40.30, 83.06, 83.82, 99.99, 117.08, 117.69, 124.12, 124.29, 135.41, 135.56, 136.12, 149.13, 149.22, 149.40, 162.81, 162.95. Analysis calculated for C_20_H_16_N_6_O_3_ (388.38): C, 61.85; H, 4.15; N, 21.64; O, 12.36. Found: C, 61.84; H, 4.14; N, 21.63.

#### ***Synthesis of pyrido[2,1-b]***[1,3,4]***oxadiazine-dialdehyde chitosan*** (7)(PODACs)

In a bottle 250 mL, 5 g of chitosan was dissolved in 125 mL acidified distilled water with HCl (2%) and stirred to obtain a complete homogenous solution. 46 mmol of sodium metaperiodate (NaIO_4_) was added at acidic pH and the reaction container was covered with aluminium foil to avoid photo-induced decomposition of the periodate. The reaction was stopped after 24 h by adding 30 ml of ethylene glycol. The resulting dialdehyde chitosan (DACs) was washed with ethanol and dried at room temperature [14]. In a mild reaction, PODACs was synthesized from the reaction of DACs (6) and PO (1:1)(5) with continues stirring at 50^o^C for 1 h through Schiff base reaction. Finally, the product PODACs 7 was washed with ethanol many times and dried at room temperature. Compound **7** was afforded as a buff solid ^1^ H NMR (500 MHz, DMSO-*d*_*6*_): the compound not dissolved in the solvent. Analysis calculated for C_54_H_50_N_12_O_15_ (1107.05): C, 58.59; H, 4.55; N, 15.18. Found: C, 58.58; H, 4.53; N, 15.17.

#### Antimicrobial activity

Antimicrobial activity of the prepared composite and starting material was evaluated against Gram-negative bacteria (*Escherichia coli* ATCC25922), Gram-positive bacteria (*Staphylococcus aureus* ATCC25923 & *Bacillus subtilis* ATCC6051), unicellular fungi (*Candida albicans* ATCC90028). Bacterial strains were cultured on nutrient agar at 37 °C for 24 h., while fungal strains were inoculated on PDA plates then incubated for 3–5 days at 28 ± 2 °C; and then kept at 4 °C for further use [15–17]. The diffusion test in agar was performed following the document M51-A2 of the Clinical Laboratory Standard Institute [18] with minor adaptations. The selected bacterial strains were cultured on nutrient agar media for 24 h at 37 °C. Bacterial suspensions of 1.5 × 10^6^ CFU/mL were separately prepared, seeded into Muller Hinton agar media, and poured aseptically into sterilized petri plates. 100 µl of each sample (composite, starting material, ampicillin/sulbactam as standard antibiotic) at concentration 1000 µg/ml was added in agar well individually, and then plates were put in the refrigerator for 2 h followed by incubation at 37 °C for 24 h. On the other hand, fungal strains were initially grown on PDA plates and incubated at 30 °C for 3–5 days [19, 20]. The fungal suspension was prepared in sterilized phosphate buffer solution (PBS) pH 7.0; the inoculums were adjusted to 10^7^ spores/ mL after counting in a cell counter chamber. One ml was uniformly distributed on agar PDA Plates. Sterile cork borer (8 mm) was used for making well in inoculated PDA plates, and then 100 µl of each sample (composite, starting material, Amphotericin B as standard antifungal drug) at concentration 1000 µg/ml was added individually. All PDA plates were incubated at 30 °C for 72 h; the inhibition zone diameter was measured. Additionally, the minimum inhibitory concentration (MIC) was determined according to the microdilution method in agar diffusion. Different concentrations for each compound (1000–7.81 µg/ml) were performed to determine MIC [21].

#### Cytotoxicity

The cytotoxicity of composite and starting material was determined using the MTT protocol [22, 23] with minor modification. The normal Vero cell lines and MCF7 (breast cancer) cell lines were collected from the American type culture collection (ATCC). The cell quantity and the percentage of the viable cell were totaled by the following formula:

$$\mathbf{V}\mathbf{i}\mathbf{a}\mathbf{b}\mathbf{i}\mathbf{l}\mathbf{i}\mathbf{t}\mathbf{y} \mathbf{\%} = \frac{\text{T}\text{e}\text{s}\text{t} \text{O}\text{D}}{\text{C}\text{o}\text{n}\text{t}\text{r}\text{o}\text{l} \text{O}\text{D}} \text{X} 100$$

**Inhibition %=**100-Viability %.

#### Molecular docking

The molecular docking of PO and PODACs against Pseudomonas aeruginosa (NCID-9016) PDB(2W7Q) ,E. coli PDB:3t88), and MCF7 (breast cancer) (PDB:4xo7) has evaluated. The protein complex was fabricated using standard bond length and energy, with the Auto Dock Vina and detected by discovery Studio Client (version 4.2)[24].

## Results and discussion

### Synthesis

**Scheme 1:**
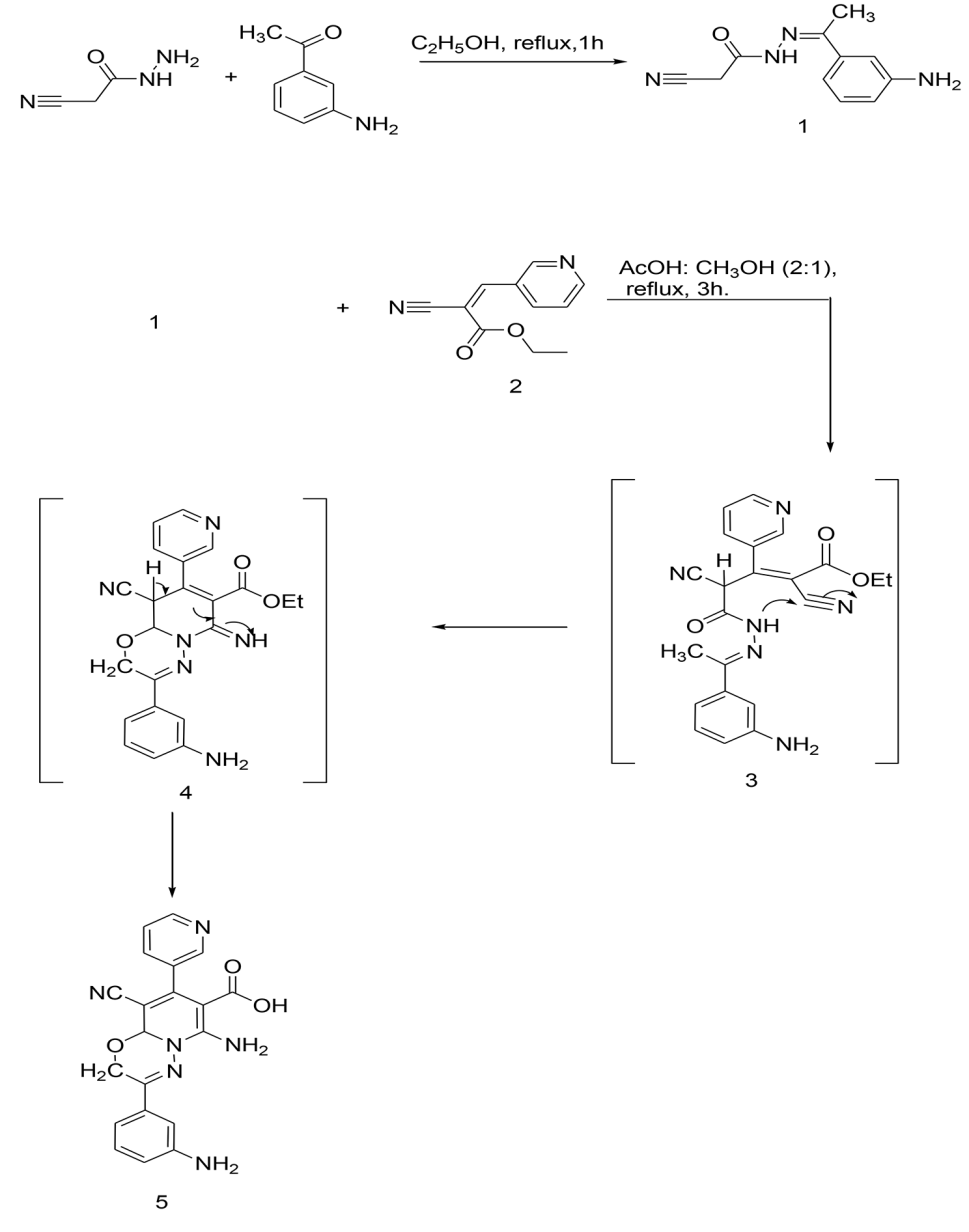
Synthesis of substituted PO

**Scheme 2:**
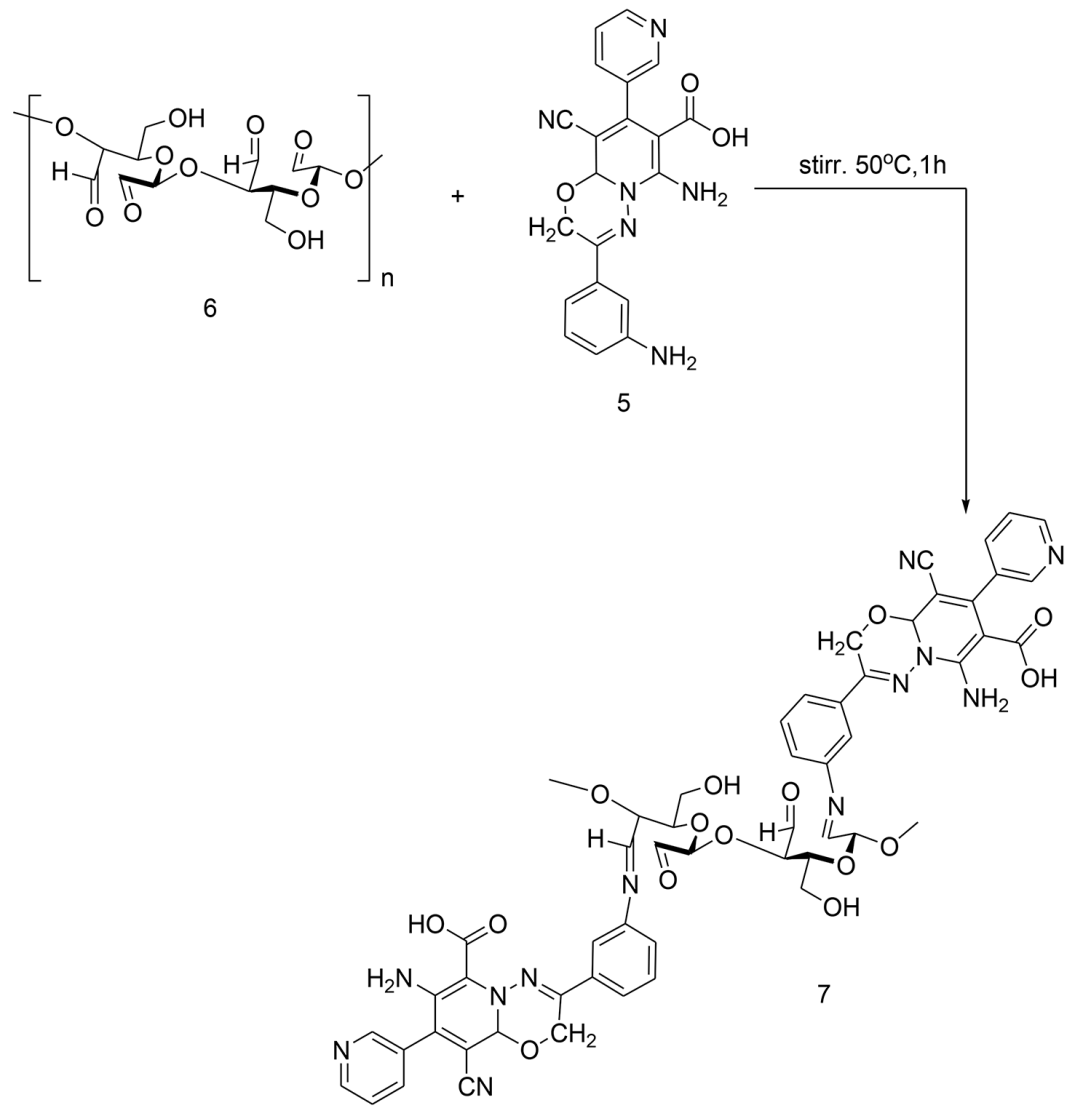
Synthesis of substituted PODACs conjugate

An approach of synthesizing the novel substituted pyrido[2,1-*b*][1,3,4]oxadiazine-7-carboxylic acid (**5**) is reported. The synthesis of the targeted structure was achieved starting from synthesizing the (Z)-*N*’-(1-(3-aminophenyl)ethylidene)-2-cyanoacetohydrazide (**1**) as a starting material by reacting the cyano acid hydrazide with the 3-amino acetophenone at reflux for hour in the presence of ethanol. The further reaction of compound **1** with the (Z)-ethyl 2-cyano-3-(pyridin-3-yl)acrylate (**2**) afforded the substituted PO (Scheme 1). The synthesis of **5** from the reaction of **1** with **2** is assumed to proceed via Michael addition of active methylene of **1** to the double bond in **2**, the formed intermediate 3 cyclized to afford 4 which undergo hydrolysis in the presence of acetic acid to afford the targeted compound 5 via the suggested mechanism as shown in scheme 1. The chemical structures of **5** were assigned on the basis of its analytical and spectral data. ^1^ H NMR spectrum of the compound exhibited a singlet signals at δ 4.47 & 4.95 ppm assignable to the protons of the methylene, and CH groups as well as signals from δ 7.35 to 8.46 ppm for eight protons residing on the two-substituted benzene rings. Additionally, the two NH2 and the OH group singlet signals are displayed at 11.00 ppm. While scheme 2. Shows Synthesis of substituted (PODACs) conjugate via the reaction of PO with DACs via Schiff base reaction. NH2 of PO has conjugated with C = O of DACs.

### Characterization

#### FTIR analysis

**Fig. 2:**
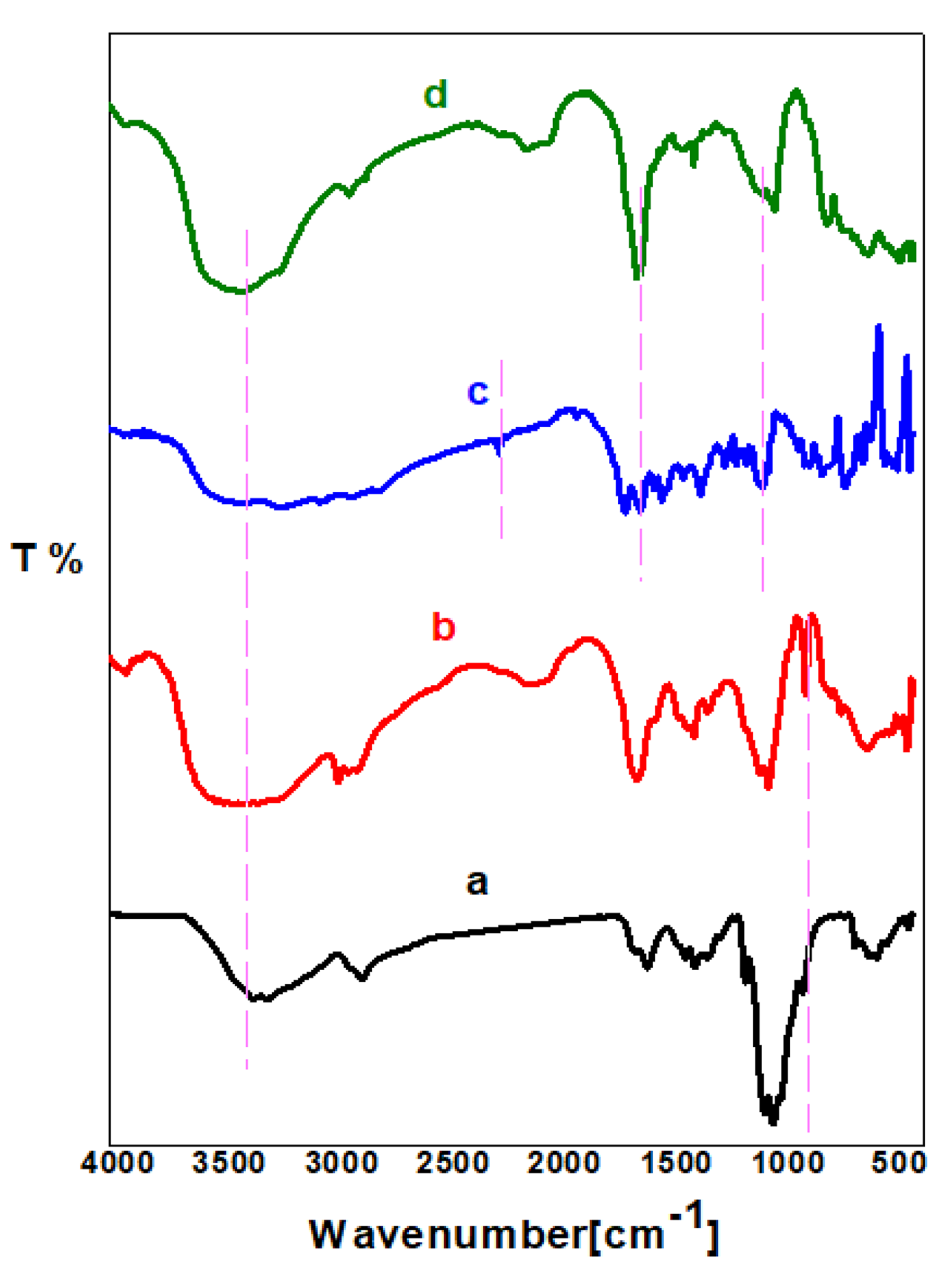
FTIR of **(a)** Cs, **(b)** DACs, **(c)** PO and **(d)** PODACs

IR analysis is a considerable technique that can be used to study the chemical structure of the prepared compounds. Figure 2 shows FTIR spectra of chitosan, DACs, PO and PODACs. The characteristic absorption peaks for chitosan were at 3300 cm^− 1^ due to the stretching vibrations of the NH_2_ and OH groups while the peak at 1650–1580 and 1100 cm^–1^ corresponding to C–N bond ether linkage respectively [25, 26]. A unique peak of DACs appears in (b) at 885 cm^− 1^ that confirms dialdehyde formation due to selective oxidation at C2- C3 of chitosan ring. In the spectra of PO derivatives (c), the peaks at 3500 cm^− 1^, 2200 cm^− 1^, 1618 cm^− 1^ corresponding to OH/ NH, CN cyano, C = N groups, respectively. While in PODACs (d), the peak of CN cyano is completely disappeared. The peaks of OH/NH, C = N and C-N due to the amino hydroxyl, amide and bond ether linkage at 3500 cm^− 1^, 1618 cm^− 1^, 1045 cm^− 1^, respectively [36].

#### Scanning electron microscope (SEM)

**Fig. 3:**
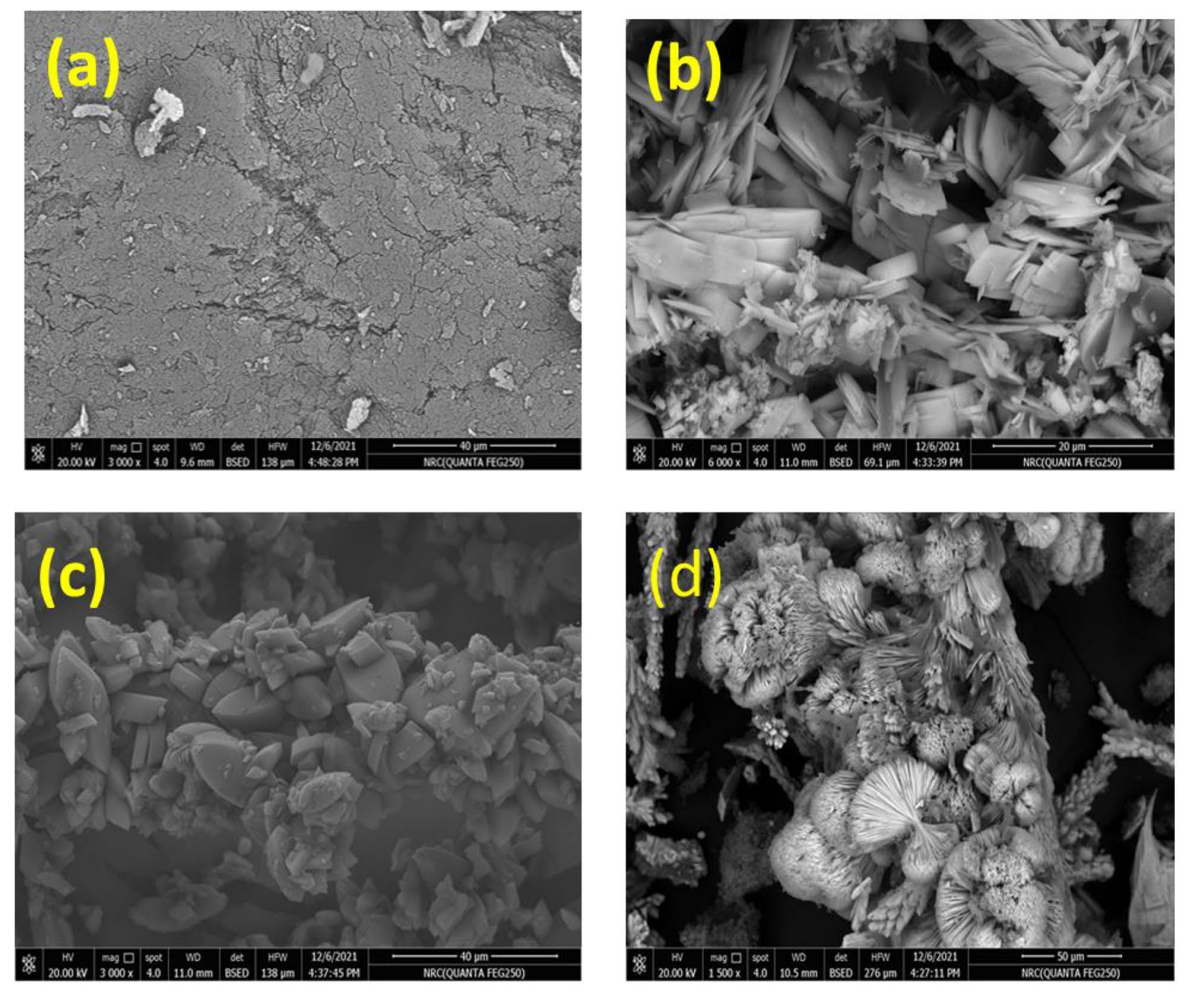
SEM of **(a)** Cs, **(b)** DACs, **(c)** PO and **(d)** PODACs

Figure 3 displays the surface morphology of chitosan, DACs, PO and PODACs. The surface morphology of pure chitosan (a) appears as a homogenous surface with some cracks. While the DACs (b) appears as a soft slice square collected in groups. The prepared PO (c) appears as a small cubic collected together in a pure crystalline surface and the final product PODACs (d) due to the reaction of DACs and PO appears as a flower- crystalline shape collected in groups.

#### X-ray diffraction

**Fig. 4:**
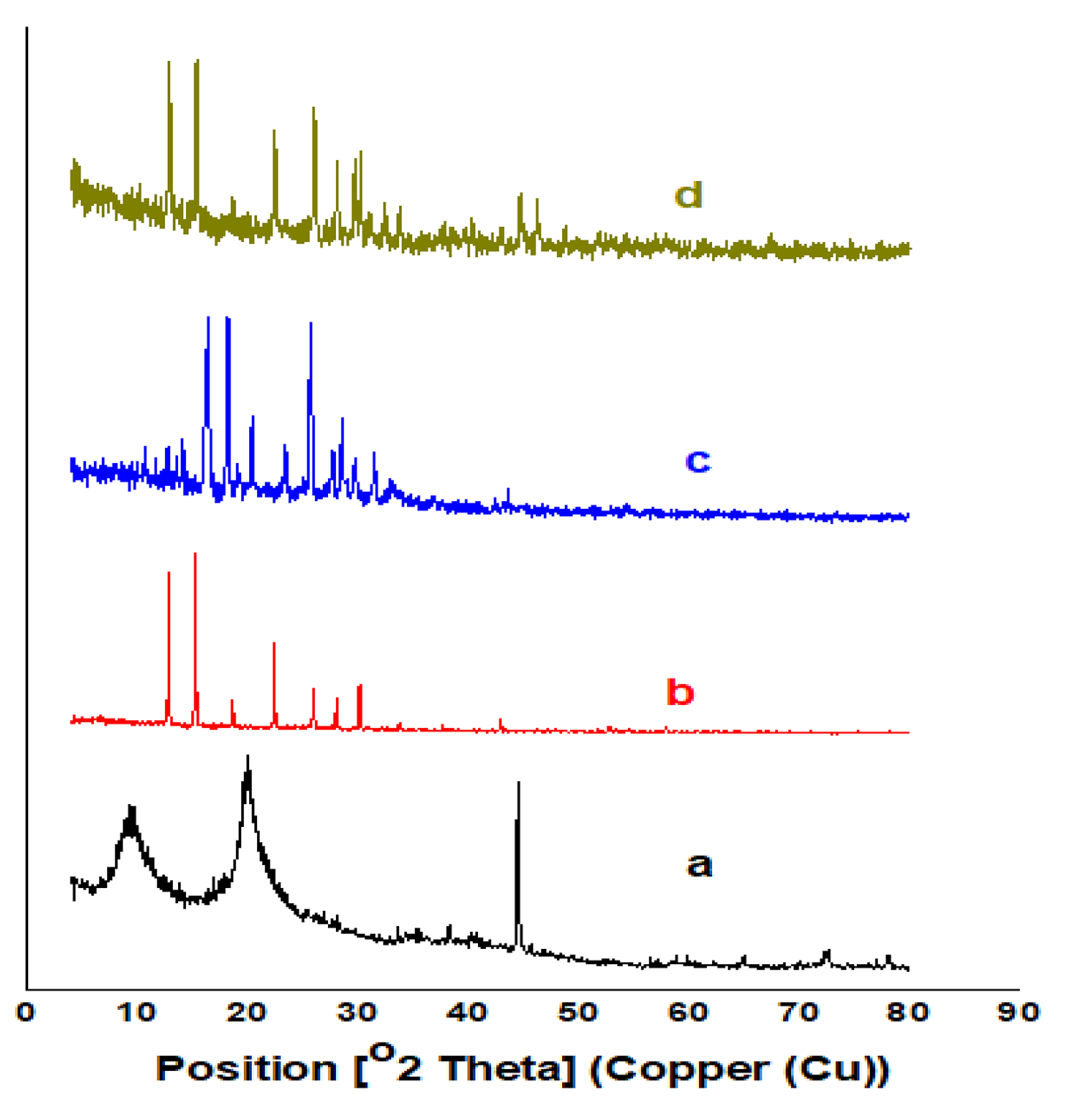
XRD of **(a)** Cs, **(b)** DACs, **(c)** PO and **(d)** PODACs

X ray is an important tool that demonstrates the amorphous and crystalline nature of different compounds. Figure 4 illustrates the amorphous and crystalline pattern of chitosan, DACs, PO and PODACs. Pattern (a) of chitosan shows three peaks at 2Ɵ=10^o^,20^o^, 45^o^ which refer to the chains of α-chitin in in the raw and crystalline region of chitosan [27] while the pattern (b) shows DACs after oxidation which has been converted to the crystalline form that appears as a sharp peaks at 2Ɵ= 15^o^-35^o^. The pattern (c, d) represent a crystalline substituted PO and c) PODACs with sharp peaks 2Ɵ= 18^o^-35^o^ [38].

#### Molecular docking

**Fig. 5:**
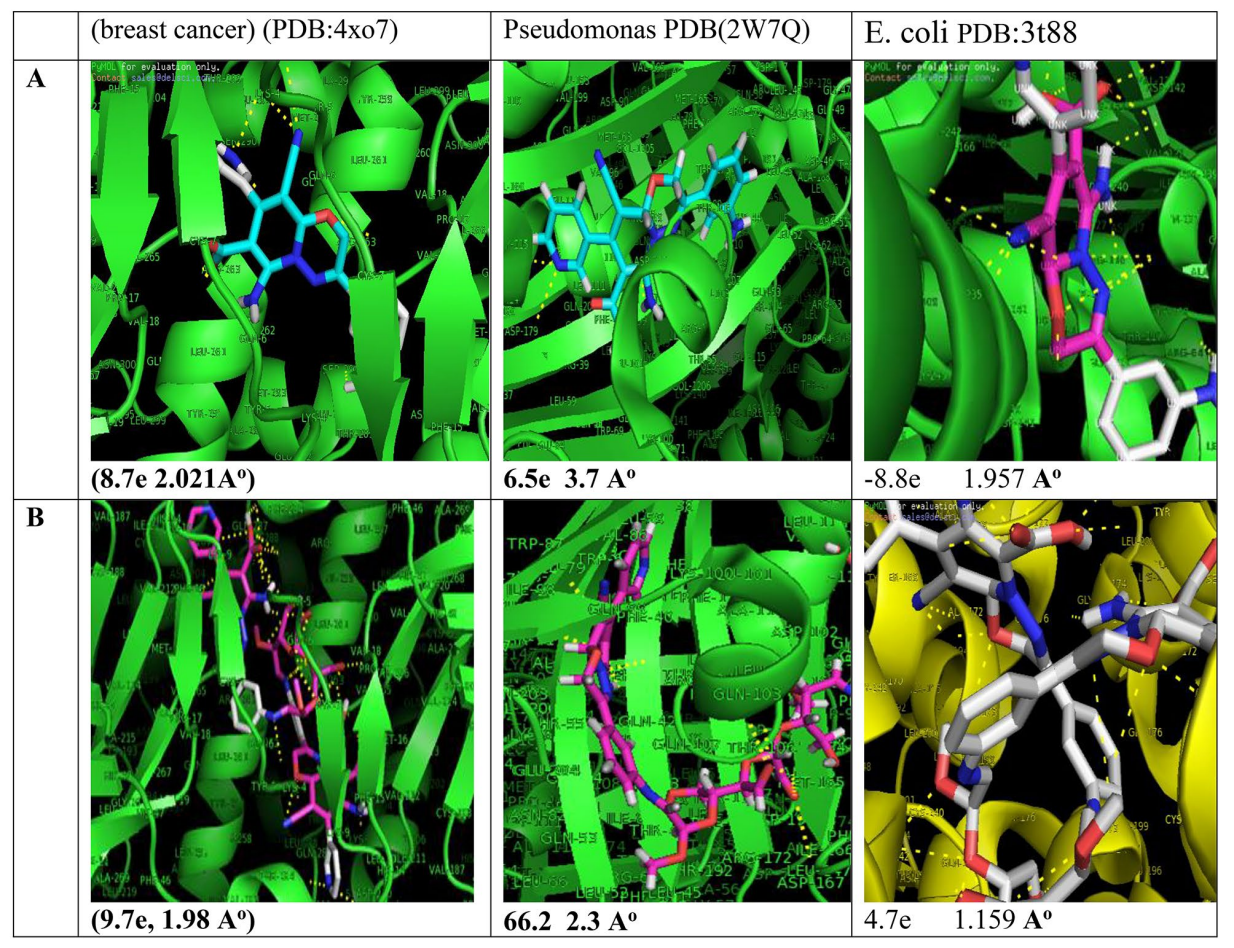
Molecular docking of **(A)** PO and **(B)** PODACs

The interaction between the ligand and protein is confirmed by studying the bond length and the energy. As the bond length decrease as the ligand became near to the protein pocket and can effect on this protein. Figure 5 shows molecular docking of PO (A) and **PODACs***(B)* against Pseudomonas aeruginosa (NCID-9016) PDB(2W7Q), E. coli PDB(3t88), and MCF7 (breast cancer) (PDB:4xo7). As in Fig. 5 the biological activity of substituted pyrido[2,1-*b*][1,3,4]oxadiazine (A) has improved by adding *dialdehyde* chitosan as in the compound **PODACs** (B) and it appears in the bond length (**A**^**o**^) and Energy (e). where the bond length (**A**^**o**^) decreased **PODACs** (B) than the bond length (**A**^**o**^) of PO (A), which means the ligand (B) is very close to the protein pocket of bacteria and can destroy it. So it increases the biological activity [28].

### Antimicrobial activity

**Fig. 6:**
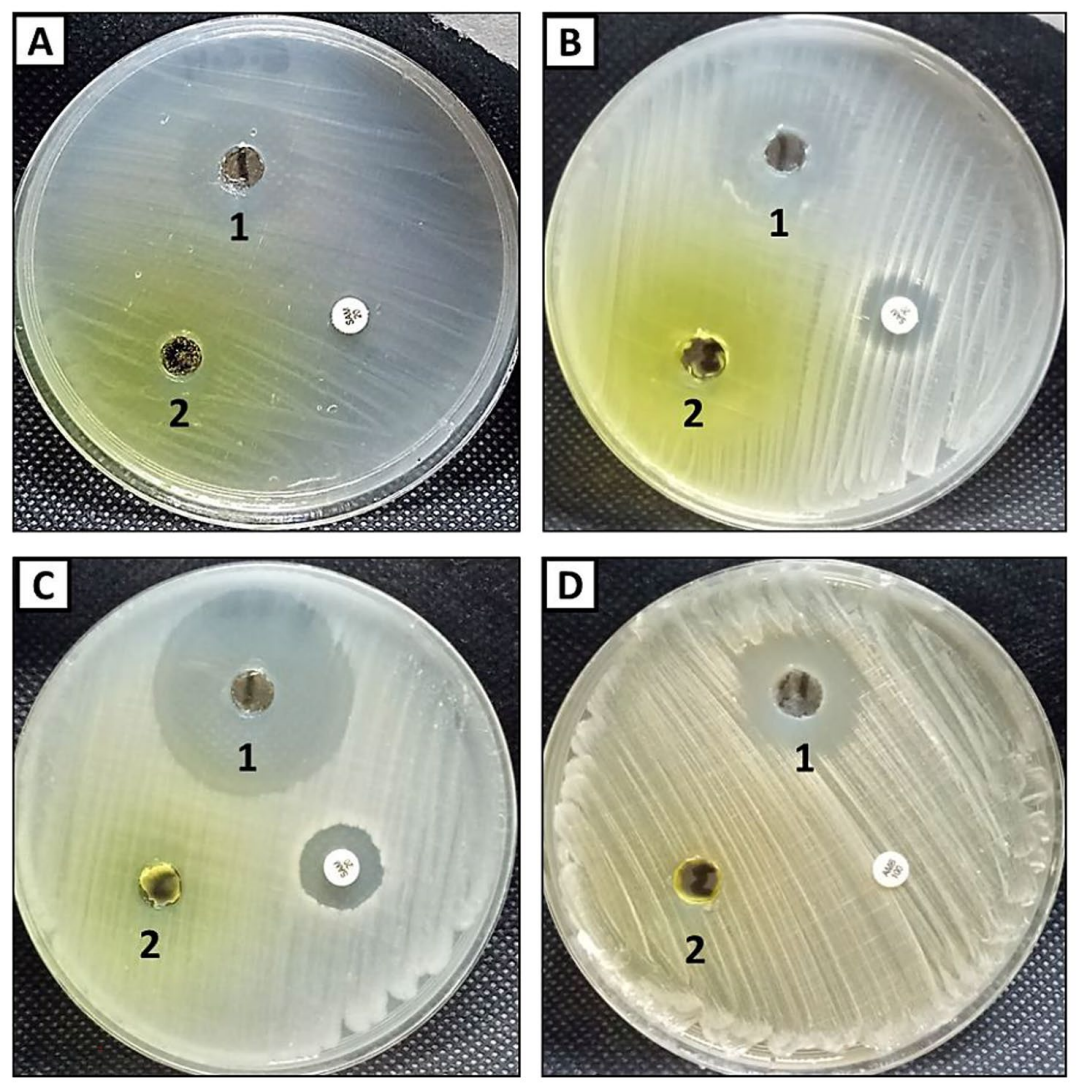
Antimicrobial activity of composite (1) and compound 5(2) against *E.coli***(A)**, *S. aureus***(B)**, *B. subtilis***(C)** and *C. albicans***(D)**

Antimicrobial activity of the prepared composite and compound 5 was evaluated against *E. coli, S. aureus, B. subtilis* and *C. albicans* using agar well diffusion method illustrated in Fig. 6A-D. Results revealed that the composite exhibited promising antibacterial as well as antifungal activity compared to compound 5 (pyrido[2,1-*b*][1,3,4]oxadiazine exhibited weak antibacterial activity against *E. coli and S. aureus* only among all tested bacterial strains, where inhibition zones were 14 and 15 mm respectively. On the other hand, pyrido[2,1-*b*][1,3,4]oxadiazine did not exhibit any activity neither *B. subtils* nor *C. albicans*. Previous studies confirmed that ][1, 3, 4]oxadiazine have antibacterial and antifungal activities but some of bacterial and fungal strains are resistant to it [29, 30]. Therfore, in this study dialdehyde chitosan was added to pyrido[2,1-*b*][1,3,4]oxadiazine as a try to increase antimicrobial activity. Results illustrated that, addition of dialdehyde chitosan to pyrido[2,1-*b*][1,3,4]oxadiazine led to significant increase in its antibacterial and antifungal activity against all tested bacterial and fungal strains. This composite (pyrido[2,1-*b*][1,3,4]oxadiazine-dialdehyde chitosan) exhibited promising antibacterial activity against Gram-negative bacteria (*E. coli*) and Gram-positive bacterial (*S. aureus* and *B. subtilis*) where inhibition zones were 19, 18 and 36 mm respectively. Likewise, MIC of the composite was in accordance with inhibition zone, where MICs were 125, 125 and 15.62 µg/ml toward *E. coli, S. aureus* respectively (Table 1. Furthermore, the composite gave antifungal activity against *C. albicans* where inhibition zone was 20 mm, whileas MIC was 62.5 µg/ml. On the other hand standard antibiotic/antifungal exhibited weak antimicrobial activity against Gram-positive only, but did not exhibit any activity toward Gram-negative bacteria. Moreover, *C. albicans* was resistant to standard antifungal (AMB) where AmB did not exhibit any activity. Therefore, the prepared composite in the current study can be used as a new antimicrobial agent in biomedical field [37].

**Table 1:** Inhibition zone and MIC of composite against bacterial and fungal strains

Bacterial/fungal strain	Composite		Compound 5		SAM /AMB
	IZ	MIC	IZ	MIC	
***E.coli***	19	125	14	500	ND
***S. aureus***	18	125	15	500	12
***B. subtilis***	36	15.62	ND	ND	15
***C. albicans***	20	62.5	ND	ND	ND

### Anticancer activity

**Fig. 7:**
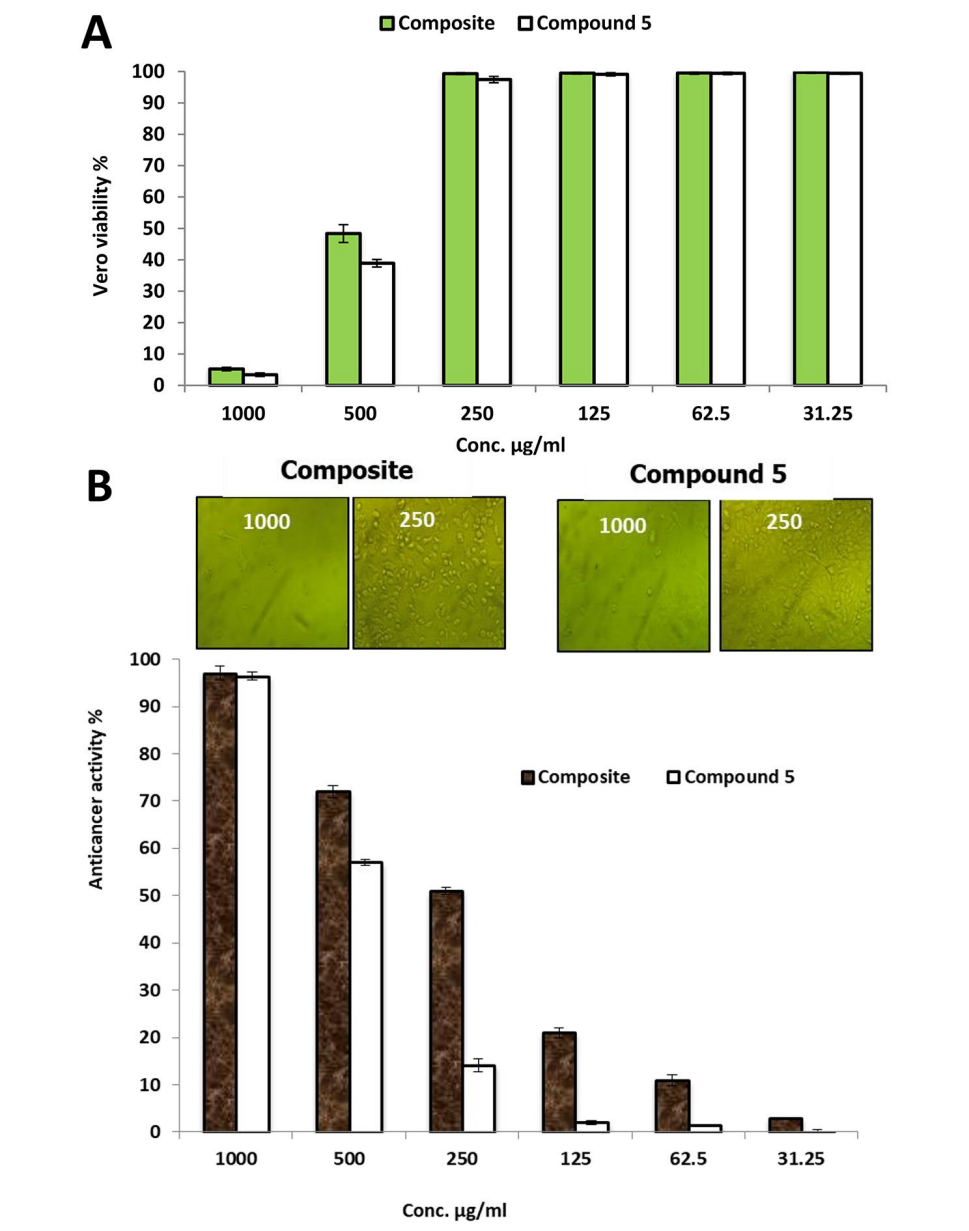
Cytotoxicity of composite and compound 5 on vero normal cell line **(A)** and Cancerous MCF7 cell line **(B)**

Generally, evaluation the cytotoxicity of new compounds on in vitro human normal cell lines is the first step to detect their safety for human use [23]. The cytotoxicity of the prepared composite and compound 5 toward Vero normal cell line were evaluated as illustrated in Fig. 7A; Table 2. Results revealed that IC_50_ of the prepared composite (pyrido[2,1-*b*][1,3,4]oxadiazine-dialdehyde chitosan) and compound 5 (pyrido[2,1-*b*][1,3,4]oxadiazine) toward Vero normal cell line was 472 and 419 µg/ml. Also, result showed that viability of Vero cells at different concentrations of composite 250, 125, 62.5 and 31.25 µg/mL was more than 99% for each. In general, if the IC_50_ is ≥ 90 µg/mL, the material is classified as non-cytotoxic [31].

Anticancer activity of compound 5 (pyrido[2,1-*b*][1,3,4]oxadiazine) and composite (pyrido[2,1-*b*][1,3,4]oxadiazine-dialdehyde chitosan) at concentrations ranged from 1000 to 31.25 µg/mL was carried out against cancerous cell lines MCF7 as shown in Fig. 7B. Results illustrated that pyrido[2,1-*b*][1,3,4]oxadiazine without dialdehyde chitosan exhibited weak anticancer activity where IC_50_ was 405 µg/ml, but pyrido[2,1-*b*][1,3,4]oxadiazine-dialdehyde chitosan exhibited anticancer activity against MCF7 higher than pyrido[2,1-*b*][1,3,4]oxadiazine only where IC_50_ was 238 µg/ml as shown in Table 2. In a previous study, 1,3,4-oxadiazine pyran derivatives exhibited antitumor activity against breast adenocarcinoma (MCF-7), non-small cell lung cancer (NCI-H460) and CNS cancer (SF-268) [32]. Many previous studies confirmed the anticancer activity of 1,3,4-Oxadiazole [33–35].

**Table 2:** Cytotoxicity of composite and compound 5 on vero normal and Cancerous MCF7 cell lines

ID	Conc µg/ml	VERO		MCF7	
		Viability %	IC50 (µg/ml)	Toxicity %	IC50 (µg/ml)
**Composite**	1000	5.2 ± 0.55	472	97.04 ± 1.4	238
	500	48.36 ± 2.8		72 ± 1.3	
	250	99.32 ± 0.3		51 ± 0.7	
	125	99.45 ± 0.3		21 ± 1.0	
	62.5	99.50 ± 0.4		11 ± 1.2	
	31.25	99.72 ± 0.3		3 ± 0.01	
**Compound 5**	1000	3.39 ± 0.55	419	96.3 ± 0.8	405
	500	38.90 ± 1.2		57 ± 0.55	
	250	97.41 ± 1.0		14.16 ± 1.4	
	125	99.09 ± 0.5		2.052 ± 0.4	
	62.5	99.41 ± 0.4		1.45 ± 0.2	
	31.25	99.41 ± 0.3		0.05 ± 0.5	

## Conclusion

This work reported dialdehyde chitosan (DACs) preparation via periodat oxidation of chitosan and preparation of a new substituted pyrido[2,1-*b*][1,3,4]oxadiazine-7-carboxylic acid (PO) through reacting(Z)-*N*’-(1-(3-aminophenyl)ethylidene)-2-cyanoacetohydrazide with (Z)-ethyl 2-cyano-3-(pyridin-3-yl)acrylate. Then, a novel composite has synthesised based on substituted pyrido[2,1-*b*][1,3,4]oxadiazine-dialdehyde chitosan conjugate (PODACs). The prepared compounds have investigated via FTIR, XRD, and SEM. The prepared composite exhibited promising antimicrobial activity against *E. coli*, *S. aureus, B. subtilis* and *C. albicans*. Furthermore, the composite illustrated cytotoxic activity against MCF-7 human breast cancer cell line as well as Vero normal cell line.

## Electronic supplementary material

Below is the link to the electronic supplementary material.

Supplementary Material 1

## Data Availability

The datasets generated during and/or analyzed during the current study are available from the corresponding author on reasonable request.
