# Supplementary material for: Synthetic strategy towards novel composite based on substituted pyrido[2,1-b][1,3,4]oxadiazine-dialdehyde chitosan conjugate with antimicrobial and anticancer activities
Source: BMC Chem. 2023 Jul 26;17(1):88. doi: 10.1186/s13065-023-01005-1 (PMC10373407; doi:10.1186/s13065-023-01005-1)
Supplement: Supplementary file 1 — Supplementary Material 1 [file 13065_2023_1005_MOESM1_ESM.docx]

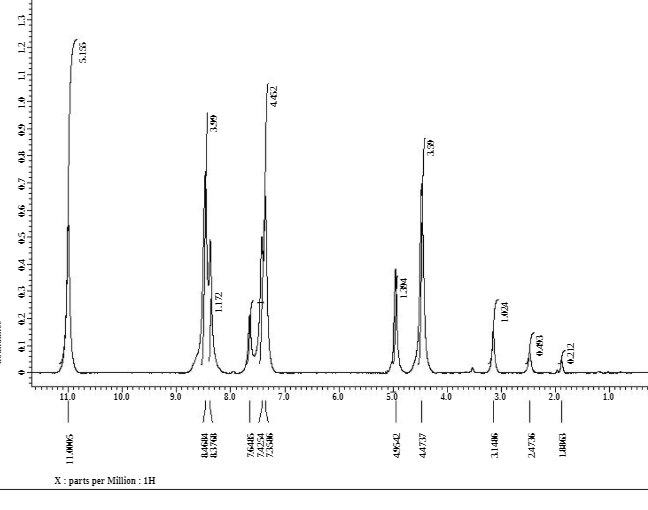


Figure S1: 1H-NMR spectrum of compound 5


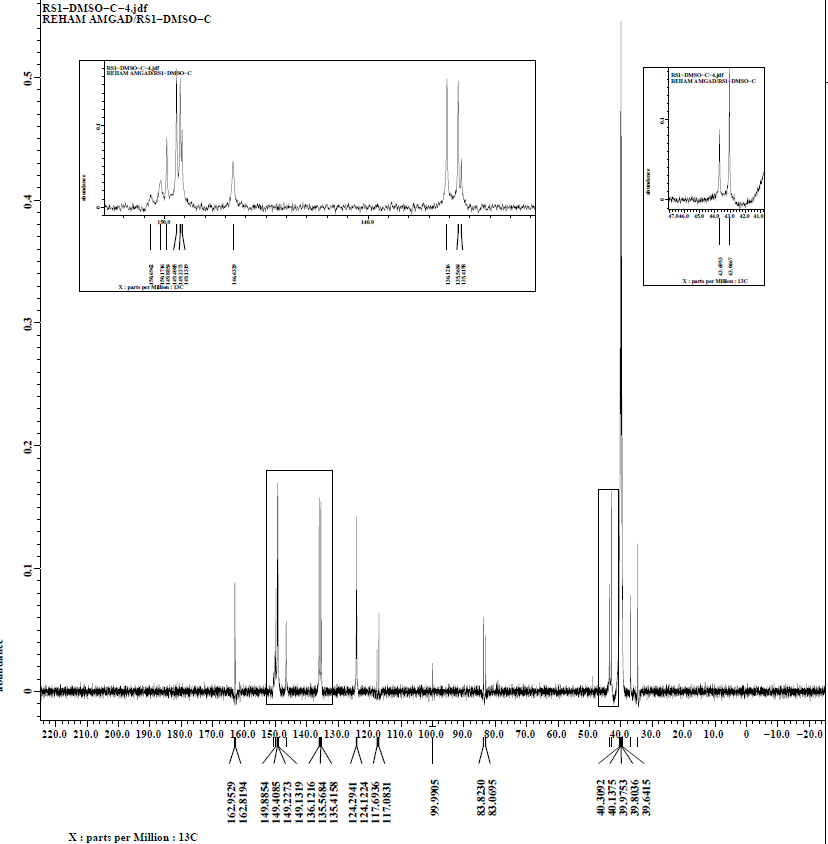


Figure S2: C^13^-NMR spectrum of compound 5


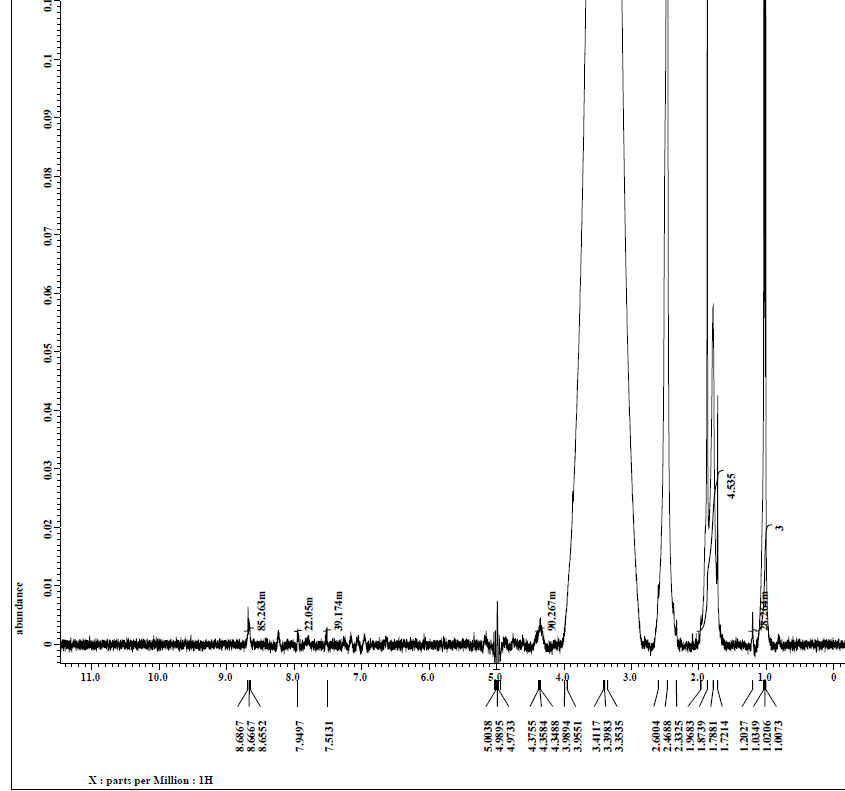


Figure S3: 1H-NMR spectrum of compound 7
